# CHARGE syndrome due to deletion of region upstream of *CHD7* gene START codon

**DOI:** 10.1186/s12881-015-0225-7

**Published:** 2015-09-03

**Authors:** Elisa Pisaneschi, Pietro Sirleto, Francesca Romana Lepri, Silvia Genovese, Maria Lisa Dentici, Stefano Petrocchi, Adriano Angioni, Maria Cristina Digilio, Bruno Dallapiccola

**Affiliations:** Medical Genetics Laboratory, Bambino Gesù Paediatric Hospital, IRCCS, Rome, Italy; Scientific Directorate, Bambino Gesù Paediatric Hospital, IRCCS, Rome, Italy; Medical Genetics, Bambino Gesù Paediatric Hospital, IRCCS, Rome, Italy; Bambino Gesù Children Hospital, Molecular Genetics Laboratory, Viale di San Paolo 15, 00146 Rome, Italy

**Keywords:** CHARGE syndrome, *CHD7* gene, CGH array, Next Generation Sequencing, deletion

## Abstract

**Background:**

CHARGE syndrome is an autosomal dominant disorder, characterized by ocular Coloboma, congenital Heart defects, choanal Atresia, Retardation, Genital anomalies and Ear anomalies. Over 90 % of typical CHARGE patients are mutated in the *CHD7* gene, 65 %–70 % of the cases for all typical and suspected cases combined. The gene encoding for a protein involved in chromatin organization. The mutational spectrum include nonsense, frameshift, splice site, and missense mutations. Large deletions and genomic rearrangements are rare.

**Case presentation:**

We report here on a 5.9 years old male of Moroccan origin displaying classic clinical features of CHARGE syndrome. Using CGH array and NGS analysis we detected a microdeletion (184 kb) involving the promoter region and exon 1 of *CHD7* gene and the flanking *RAB2* gene.

**Conclusion:**

The present observation suggests that deletion limited to the regulatory region of *CHD7* is sufficient to cause the full blown CHARGE phenotype. Different size of deletions can result in different phenotypes, ranging from a milder to severe CHARGE syndrome; this is based on a combination of major and minor diagnostic characteristics, therefore to a more variable clinical features, likely due to the additive effect of other genetic imbalances. MLPA and CGH techniques should be considered in the diagnostic protocol of individuals with a clinical suspect of CHARGE syndrome

## Background

CHARGE (MIM# 214800) is the acronym of an autosomal dominant genetic condition, characterized by ocular Coloboma, congenital Heart defects, choanal Atresia, Retardation (of growth and/or of development), Genital anomalies and Ear anomalies (abnormal pinnae and/or hearing loss, abnormal semicircular canals) [1]. *CHD7* gene has been causally linked to this disorders. The embryologic expression of the gene involves many tissues, including the eye, inner ear, and olfactory bulb cells. In addition it is widely expressed in undifferentiated neuroepithelium and in neural crest mesenchyme, and, at the end of the first trimester, in dorsal root ganglia, cranial nerves and ganglia, and auditory, pituitary and nasal tissues, as well as in neural retina [2]. Typical CHARGE patients are mutated in *CHD7* gene in over 90 % of the cases [3], 65 %–70 % for all typical and milder phenotypes combined [4–7]. The *CHD7* gene codes for one member of a family of proteins thought to play a role in the organization of chromatin, belonging to the chromodomain helicase DNA binding domain family of ATP-dependent chromatin remodeling enzymes [8]. Members of this family share a unique combination of functional domains consisting of two N-terminal chromodomains, followed by a SWI2/SNF2-like ATPase/helicase domain and a DNA binding domain [9, 10]. It is assumed that CHD protein complexes affect chromatin structure and gene expression, thus playing an important role in regulating embryonic development. The CHD7 protein most likely controls gene expression by chromatin remodelling. Chromatin remodelling is the dynamic modification of chromatin architecture, allowing the access of condensed genomic DNA to the regulatory transcription machinery proteins. Gene expression is lowered in the presence of tight DNA packaging.

CHARGE syndrome is caused by heterozygous mutations, including nonsense mutations (44 %), frameshift mutations (34 %), splice site mutations (11 %) and missense mutations (8 %) [11]. Deletions and genomic rearrangements occur in 3% of the cases. Variants expected to lead to a truncated protein (nonsense and frameshift mutations and deletions) are considered to be pathogenic as a consequence of haploinsufficiency [12].

We report here on a male patient affected by CHARGE syndrome, heterozygous for a deletion involving the promoter region and exon 1 of the *CHD7* gene and the contiguous *RAB2* gene.

## Case presentation

The patient, a male of Moroccan origin, is the first child of healthy unrelated patients. Family history was unremarkable. At birth, the mother was 22 years old, the father 26. The baby was born at the 41 week of an uneventful gestation. Birth weight was 2740 g, length 47 cm, head circumference 32.5 cm. Apgar scores were 7 and 8 at 1 and 5 minutes. Neonatal period was complicated by respiratory distress. Congenital heart defect was diagnosed in the first day of life. Two-Dimensional color-Doppler echocardiography showed patent ductus arteriosus (PDA) and retroesophageal right subclavian artery. PDA was treated by interventional catheterization. Bilateral vesico-ureteric refluxes was diagnosed by renal ultrasound and cystography, and operated endoscopically at age 1 and 2 years. Ophthalmological examination demonstrated bilateral ocular coloboma of iris, retina and optic nerve and right eye microphthalmia. Horizontal bilateral nystagmus was also present. Brain-stem-evoked audiometry showed profound bilateral sensorineural hearing loss, prompting cochlear implants positioning at 18 months of age. Hypoplastic semicircular canals were diagnosed by temporal bone CT-scan. Cerebral ultrasound and electroencephalogram were normal.

Developmental milestones were delayed. The patient sat at 15 months, and started walking at 28 months. Language was absent. Feeding difficulties were recorded, resulting in growth deficiency. Bilateral cryptorchidism was operated at 2 years.

At time of last evaluation the patient was 5.9 years old. Weight was 13,9 kg, height 102 cm, head circumference 46.5 cm (all paramerters below the 3^rd^ centile). Clinical features included microcephaly, facial palsy, right microphthalmia, down-slanting palpebral fissures, prominent nose with round tip, everted upper lip, multiple dental caries, micrognathia, cupped asymmetric and low-set ears, dysplastic toe nails.

## Materials and methods

Array comparative genomic hybridization (array-CGH) was performed with the Agilent Human Genome CGH Microarray 60K kit (Agilent Technologies, Santa Clara, CA, U.S.A.). This platform is a 60-mer oligonucleotide-based micro- array that allows a genome-wide survey and molecular profiling of genomic aberrations with a resolution of about 41 kb. DNA was extracted from peripheral blood using QIAampH DNA Blood Kit (QIAGEN Sciences, Germantown, MD, U.S.A.) according to the manufacturer’s instructions (Agilent Oligonucleotide Array-Based CGH for Genomic DNA Analysis – Version 7.2, July 2012). The array was analyzed through the Agilent Scanner and the Feature Extraction software (v10.7.3.1) and Agilent Genomic Workbench 7.0.4. Bioinformatic analysis was carried out by consulting the Database of Genomic Variants BioXRT [http://projects.tcag.ca/variation/]. Gene content analysis in the deleted segment was carried out by using UCSC database NCBI37/hg19 (http://www.genome.ucsc.edu).

Next Generation Sequencing analysis was performed on DNA extracted from peripheral blood of the patient. The *CHD7* gene is included in a panel of genes responsible for eye anomalies and was analysed by Targeted resequencing, using a uniquely customized design (TruSeq® Custom Amplicon; Illumina, San Diego, CA) with the MiSeq® sequencing platform (Illumina, San Diego, CA). TruSeq Custom Amplicon (TSCA) is a fully integrated DNA-to-data solution, including online probe design and ordering through the Illumina website sequencing assay automated data analysis and offline software for reviewing results. (According to NCBI gene: NG_007009.1; NM_ 017780; NP_060250.2).

## Results

**Fig. 1:**
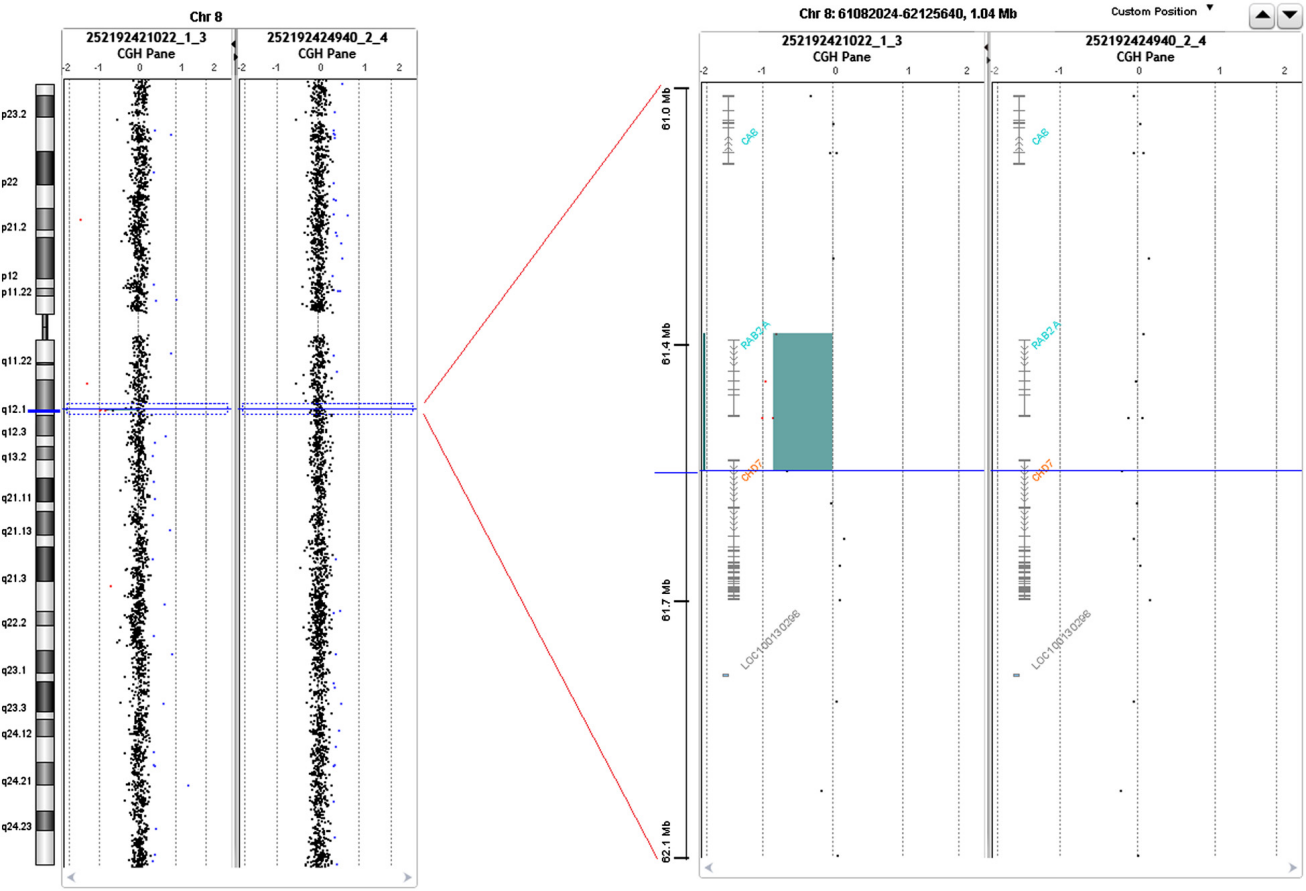
CGH array results; **a**) proband: deletion of five probes at level of RAB2A and CHD7; **b**) mother: normal

Array-CGH analysis disclosed a deletion spanning about 184 kb of genomic DNA, including *RAB2* gene (Ras-associated protein RAB2) and upstream region of *CHD7* (chromodomain helicase DNA binding protein 7) gene, including 5’UTR and untraslated exon one. Array-CGH analysis in the proband mother was normal (Fig. 1), while the father was unavailable for the study.

NGS analysis showed a variant in intron 2 (rs7836586), heterozygosity of this variant excluded deletion in this region. A fine mapping of the deletion revealed that one breakpoint was upstream of *RAB2* gene (8q12.1-61.421.463) while the second breakpoint was within intron 1 (8q12.2-61,605,551). The variant reported in the VCF output file has been evaluated for the coverage and the Qscore and visualized via Integrative Genome Viewer (IGV) [13, 14]. This analysis showed that ATG was excluded from the deletion, but important regulatory regions were lacking, including untranslated exon 1, the 5-UTR region and probably the promoter region.

## Conclusion

CHARGE syndrome is an autosomal dominant disorder, with most cases being *de novo*. The mutational spectrum of *CHD7* in patients affected by CHARGE syndrome is variable, including nonsense, frameshift, splice site and missense mutations, deletions and genomic rearrangements [11]. Rarely deletions affect the CHD7 gene, only a few cases having been reported so far: deletion of single or multiple exons, deletion of the entire gene or larger deletion that include *CHD7* gene and flanking genes. Using MLPA, Wincent et al. (2008) [15] reported microdeletions in 17 % of their patients. Palumbo et al. (2013) [16] reviewed published patients with 8q12 deletions affecting multiple genes flanking *CHD7* (Table 1).

**Table 1:** Clinical features of reported patients with 8q12 microdeletions encompassing the CHD7 gene, including the present case

Clinical feature	1	2	3	4	5	6	7	8	9
Sex	F	F	F	M	M	M	M	F	M
Ocular coloboma	+	+	+	+	+	+	-	-	+
Choanal atresia	+	-	-	-	-	-	-	-	-
External ear anomaly	+	+	+	-	+	+	+	+	+
Hearing deficit	+	+	-	+	nr	+	-	-	+
Cleft lip/palate	-	-	-	-	-	+	-	-	-
Larynx malformation	-	-	-	-	-	+	-	-	-
Facial palsy	+	-	-	-	+	+	-	-	+
Congenital heart defect	+	+	+	+	+	+	+	+	+
Genital anomaly	nr	-	+	+	+	+	-	-	+
Urinary malformation	-	-	-	-	-	+	-	-	-
Temporal bone malformation	-	+	nr	nr	-	+	-	-	+
Growth deficiency	-	+	+	nr	-	+	-	-	+
Developmental delay	+	+	+	nr	nr	+	-	-	+
Phenotype	C	C	C	C	C	C	No C	No C	C
Genetic imbalance	t(6;8)(6p8p;6q8q)	Del8q12	Del8q11.2-q13	Del ex1	Del ex4	Del CHD7	Del8q12	Del8q12.1q12.3	Del RAB2-5’UTR/ex1 CHD7

1: [17]; 2: [18]; 3: [19–21]; 4-5-6: [15]; 7: [22]; 8: [11]; 9: Present patient

C : CHARGE nr : not reported

It has been questioned if differently sized deletions can account for variable clinical expression. All known patients, but two, displayed CHARGE syndrome. Two subjects manifested severe growth retardation and failure to thrive, hypertrichosis, gastro-esophageal reflux, and neurodevelopmental defects (patients 2 and 3 in the Table 1). It has been highlighted that the clinical features of these patients in some respect overlapped those of individuals with the 8q12 duplication phenotype [23–25]: developmental delay, sensorineural deafness and a congenital renal and heart defect. This rather specific recurrent pattern of congenital anomalies associated with overlapping duplications of the genomic region containing CHD7 suggests that the phenotype may be the result of abnormal CHD7 dosage.

Potential molecular mechanisms underlying phenotypic variability of the delete CHARGE patients include deletion size, gene contents of the missing region, polymorphisms or mutations in the hemizygous allele, different genomic background or environmental factors. A dosage sensitive role of some genes, including *CA8*, *RAB2A*, *CLVS1* and *CHD7* has been also suggested [16]. The *CA8* gene product lacks carbonic anhydrase activity (i.e., the reversible hydration of carbon dioxide). The gene product continues to carry a carbonic anhydrase designation based on clear sequence identity to other members of the carbonic anhydrase gene family. The absence of CA8 gene transcription in the cerebellum of the lurcher mutant in mice with a neurologic defect suggests an important role for this acatalytic form. CLVS1 (Clavesin 1) is a Protein Coding gene; diseases associated with CLVS1 include Duane Retraction syndrome. GeneOntology annotations related to this gene include transporter activity and phosphatidylinositol-3,5-bisphosphate binding.

The present patient, presenting with a CHARGE syndrome, has a unique deletion, spanning about 184 kb of genomic DNA, including *RAB2* gene and a small part of *CHD7. RAB2A* (*RAB2A*, member RAS oncogene family) is a protein-coding gene, previously associated with vaginal cancer and vaginitis. The protein encoded by this gene belongs to the Rab family, members of which are small molecular weight guanosine triphosphatases (GTPases) containing highly conserved domains involved in GTP binding and hydrolysis. Rabs are membrane-bound proteins, affecting vesicular fusion and trafficking. This protein is a resident of pre-Golgi intermediates, and is required for protein transport from the endoplasmic reticulum (ER) to the Golgi complex. To identify the role of Rab2 in membrane trafficking, Tisdale et al (1992) [26] generated site-directed Rab2 mutants. These mutations inhibited protein transport from the ER to the Golgi and indicate that Rab2 is required for ER to Golgi trafficking. Spliced transcript variants encoding different isoforms are known (provided by RefSeq, Oct 2011). To date, there is no evidence of any link between *RAB2* mutations and CHARGE syndrome, but we can not exclude a role of the deletion on the final phenotype of our patient.

Most of the phenotype of our patient is likely due to the deletion of the 5’UTR region and exon 1 of *CHD7* with consequent block of gene translation. Sequencing analysis did not reveal any additional pathogenic variant in *CHD7* gene, but some polymorphisms acting as possible phenotypic modifiers (heterozygous SNPs: rs7836586, rs4540437, rs10448027, rs6471902, rs138947382, rs7005873, rs7844902 and rs139382713).

In conclusion, small deletions including the *CHD7* gene result in CHARGE syndrome. Larger deletions can be associated with mild to severe CHARGE syndrome or a different disorder resulting from the additive effect of flanking genes. The present patient shows that deletion of region upstream of *CHD7* gene is sufficient per se to cause CHARGE syndrome. The increased number of patients diagnosed with 8q12 deletions suggests that loss of single exons is probably less rare than previously considered in these patients. Accordingly, sequencing analysis, MLPA and CGH techniques should be all considered in the diagnostic protocol of subjects with a clinical suspicion of CHARGE syndrome [3].

## Consent

Clinical investigations and genetic analyses were approved by the institutional scientific board of Bambino Gesù Children Hospital and conducted in accordance with the Helsinki Declaration. Written informed consent was obtained from the parents for publication of this case report. A copy of the written consent is available for review by the Editor of this journal.

## Abbreviations

PDA: Patent ductus arteriosus
TSCA: TruSeq Custom Amplicon
NGS: Next generation sequencing
IGV: Integrative Genome Viewer
MLPA: Multiplex Ligation-dependent Probe Amplification
CGH: Comparative genomic hybridization
